# Construction enterprises’ green financing efficiency and its influencing factors including internal and external: Based on four-stage DEA model

**DOI:** 10.1371/journal.pone.0286043

**Published:** 2023-06-08

**Authors:** Yaguai Yu, Panyi Shen, Yina Yan, Taohan Ni, Fangyuan Chen

**Affiliations:** 1 Business School, Ningbo University, Ningbo, China; 2 Donghai Academy, Ningbo University, Ningbo, China; 3 Business School, University of Nottingham, Ningbo, China; Nanjing Audit University, CHINA

## Abstract

Under the background of carbon neutrality, green development is the theme of today’s times. The construction industry is an important part of the green development plan, and it is of great significance to study its green financing efficiency. Based on this, this paper uses the four-stage DEA model to explore the green financing efficiency of listed construction companies from 2019 to 2020. The conclusion shows that: firstly, the green financing efficiency of listed construction companies is low, and the demand for green financing has not been met. It is necessary to strengthen the support of green finance to meet the needs of its expansion. Secondly, the efficiency of green financing is significantly and complexly affected by external influencing factors. It is necessary to dialectically treat external influencing factors such as local industry development support, financial development level, and the number of patent authorizations. Thirdly, among the internal influencing factors, the proportion of independent directors has a significant positive impact on the green financing efficiency of listed construction companies, and the proportion of R&D investment has a significant negative impact. Listed construction companies need to increase the proportion of independent directors and control the proportion of R&D investment.

## Introduction

In September 2020, President Xi Jinping announced at the general debate of the 75th United Nations General Assembly: "China’s carbon dioxide emissions will strive to peak before 2030, and strive to achieve carbon neutrality before 2060." A major strategic decision made in response to the responsibility of a community with a shared future for mankind and the realization of the inherent requirements of sustainable development. To achieve carbon neutrality, decarbonization must be accelerated at the level of the real economy.

The construction industry is a pillar industry in China. For any country or region, an important symbol of industrial modernization is the development of its construction industry. And the degree of development of the construction industry can also reflect the degree of local economic development. However, while promoting the rapid growth of the national economy, the construction industry consumes a large amount of construction materials, which has a negative impact on the environment. And the contradiction between economic growth and environmental quality is increasing. According to the "China Building Energy Consumption Research Report (2020)", the construction industry consumes more than 40% of China’s total energy consumption, and its emission reduction potential is as high as 74%, ranking first among the three major energy consumption sectors [1]. In 2013, General Office of the State Council of the People’s Republic of China issued a "Green Building Action Plan", and the local government respond positively. In 2019, the National Development and Reform Commission issued the "Green Industry Guidance Catalogue" to promote the industrialization of green buildings. In 2020, seven ministries including the Ministry of Housing and Urban-Rural Development jointly issued the "Action Plan for the Creation of Green Buildings" requiring vigorous development of prefabricated buildings such as steel structures, and steel structures in principle for new public buildings. In 2021, the Ministry of Housing and Urban-Rural Development formulated the "Green Building Label Management Measures", and green buildings will move towards standardized development. In 2021, the Ministry of Housing and Urban-Rural Development issued the "Green Construction Technical Guidelines (Trial)" to further clarify the overall requirements, main objectives and technical measures of green construction. Taking the road of greening the construction industry has become an inevitable requirement to achieve the goal of carbon neutrality. However, due to the positive externalities of green building projects, coupled with a series of problems such as market information asymmetry and lack of credit system, market-based financing of green building projects still faces many obstacles, and the support of green finance to provide green financing is urgently needed.

China began to vigorously promote the construction of a green financial system in 2015. As the country has moved from the advocacy stage to the substantive promotion stage, green finance has become a new channel for the financing of listed companies in the construction industry, and gradually establish the green finance theory with Chinese characteristics [2]. In 2016, seven ministries including the People’s Bank of China jointly issued the "Guiding Opinions on Building a Green Financial System". It gives the official definition of green finance in China for the first time: "financial services provided to support economic activities such as environmental improvement, response to climate change and resource conservation and efficient use, namely, project investment and financing, project operation, risk management, etc. in the fields of environmental protection, energy conservation, clean energy, green transportation, green buildings, etc." Meanwhile, it can mobilize and motivate more social capital to invest in green industries. Based on this, with environmental factors as the financing object, the demander obtains financing through green financial instruments, thus promoting the rise of green financing for green development. Since then, many ministries and industry associations promote and encourage the use of green financial tools to support green building projects. Green buildings have been included in the support category of China’s green financial system. However, the development of green finance in the construction industry is still lagging behind. Although various financial tools exist to support the green building field, it is still difficult to meet the multi-level and diversified green financing needs of the green development strategy of the construction industry [3].

In the context of carbon neutrality, the reduction of carbon emissions is imminent and has great potential. Green development has become an inevitable trend in the construction industry. But there is a huge funding gap in the green development of the construction industry, which is still difficult to meet the green financing needs of the construction industry. Looking further, the construction industry still has the pain point that the efficiency of green financing is generally not high, that is, the construction industry enterprises have poor ability to integrate green funds at the lowest cost and risk and use the integrated green funds to create benefits, and fail to achieve the optimal input and output of green funds, making the green financing situation of the construction industry more severe. Therefore, it is of great significance to study the green financing efficiency of listed construction companies.

To sum up, based on the green finance theory with Chinese characteristics and the input-output efficiency theory, based on the current development situation of green finance with Chinese characteristics, this paper takes the input-output efficiency analysis as the core analysis idea, and takes the listed companies in China’s construction industry in 2019–2020 as the sample to analyze their green financing efficiency and internal and external influencing factors. The research of this paper is mainly divided into three steps: firstly, construct a four-stage DEA model and select relevant indicators to lay the research foundation. Secondly, calculate the green financing efficiency of listed companies in the construction industry and verify the impact of the selected internal and external factors. Finally, the research conclusions and suggestions are drawn.

This paper uses the four-stage DEA model to analyze the green financing efficiency and the internal and external influencing factors of listed companies in the construction industry. The possible innovation points are as follows: firstly, unlike most literatures focusing on the financial field, this paper takes the construction enterprises as the research object, analyzes the current situation of their green financing efficiency and the possible influencing factors, and enriches the research literature of green financing efficiency. Second, introduce the green finance theory with Chinese characteristics and the input-output efficiency theory at the theoretical level, and use the input-output analysis idea to more intuitively analyze the current situation of green financing efficiency of construction enterprises under the background of Chinese characteristics, so as to further expand the research case of the green finance theory system with Chinese characteristics.

## Literature review

There is currently no unified definition of green financing efficiency, and it is an expansion of research related to green finance. Jose Salazar earlier proposed green finance as a necessary financial innovation that seeks a path to environmental protection and has a connecting effect on the financial and environmental industries [4]. Based on previous studies, Ma, Ning and Zeng et al. looking forward to the development of green finance, call the use of green credit, green bond market, green stock index and related products as green financing, the effectiveness of green financing is green financing efficiency [5–7].

On the basis of confirming the connotation of green financing efficiency, scholars have launched a multi-faceted investigation into the issue of green financing efficiency. Some scholars take the perspective of the input side of green finance, Lian et al. take the banking industry that implements green credit as the research object, the study concludes that the overall efficiency of the banking industry in China in carrying out green credit is not high, and the government should strengthen their green credit incentivizing policies to promote the coordinated development of green finance and a green economy [8]. Liu et al. takes the green credit policy in 2012 as a quasi-natural experiment, and finds taht the implementation of "green credit guidelines" policy has significantly improved the green innovation performance of high-polluting and high-energy consuming enterprise [9]. Some scholars, on the other hand, take the green finance investee as the research object. Zhang et al. used the entropy value method and DEA-Malmquist index to measure the green finance efficiency at the national, inter-provincial and regional levels, using green credit, green securities and other green finance funds as input indicators, the net profit of enterprises to represent the economic contribution and the amount of tax paid by enterprises to represent the social contribution, and found that most regions’ current green financing efficiency is low [10].

In addition to measuring green financing efficiency, scholars also focus on the influencing factors of green financing efficiency. Yu et al., Lee and Lee argue that the influencing factors of enterprise financing efficiency can generally be divided into two categories: external influencing factors and internal influencing factors of enterprises, and the same applies to green financing efficiency [11, 12]. In the study of external influencing factors, Lin et al. used the environmental protection industry as the research object and established a multiple linear regression model to empirically find that the level of financial development has a significant impact on the efficiency of green financing [13]. Cao et al. took heavy polluters and green enterprises as the research objects then found empirically that the financing efficiency of heavy polluters decreased and the green financing efficiency of green enterprises increased under the green finance policy [14]. On the other hand, Qiao et al. selected provincial green public companies as the sample and used the panel regression model, found that the level of economic development, the level of scientific and technological innovation, and the regional financial environment had a significant positive impact on the efficiency of green financing in the provinces along the route [15]. In the study of internal influencing factors, Lei and Liu used the tobit regression model, found that the proportion of corporate independent directors, current debt ratio, and employee compensation are significantly and positively related to the financing efficiency of green low-carbon companies [16]. Based the data of listed energy-saving and environmental protection companies of China from 2010–2019, Jin et al. also used the tobit regression model, found that firm’s size and debt ratio have an impact on the green financing efficiency of firms [17]. Zhang et al. uses difference in difference (DID) method and finds Green credit policy could affect the financing cost of corporate debt by changing the financing scale and commercial credit [18].

In summary, scholars have achieved significant results around the measurement of green financing efficiency and the analysis of influencing factors, which provide a rich theoretical basis for the study of this paper. However, it is not difficult to find that there are few studies on green financing efficiency with construction industry as the evaluation object. The measurement index system currently used by scholars has strong authority, but it is not suitable for construction companies direct measurement and evaluation of green financing efficiency. The external influencing factors of green financing efficiency are relatively consistent, but there is no consensus on the internal influencing factors. Based on this, this paper uses the panel data of 41 listed construction companies from 2019 to 2020, and the four-stage DEA model to measure and analyze their green financing efficiency and influencing factors, so as to provide a scientific basis for improving the green financing efficiency of listed construction companies.

## Research design

### Introduction of four-stage DEA model

DEA (Data envelopment analysis) is a method of operational research and research on economic production boundary. This method is generally used to measure the production efficiency of some decision-making departments. DEA research is relatively mature, since its inception is widely used in the calculation of financing efficiency. Ali and Lerme, Panwar et al. argue that DEA has the following advantages: firstly, DEA is particularly suitable for problems with multiple inputs and multiple outputs; secondly, DEA is a non-statistical and non-parametric method that neither requires a specific functional form nor makes assumptions about the random characteristics of the data; thirdly, there is no requirement for weights, and only the actual input and output data of the decision unit are used to find the optimal weights, which has a strong objectivity [19, 20].

The inefficiency of general enterprises come from three sources: (1) inefficiency caused by the enterprise itself; (2) inefficiency caused by the influence of the external environment in which the enterprise is located; (3) inefficiency caused by the influence of random disturbances. Three-stage DEA was first proposed by Fried et al., which retains the advantages of DEA, but also has the advantages of SFA, separating the interference of external influencing factors and random errors, and can more accurately estimate the efficiency of different production units, more closely matching the real operating conditions of enterprises [21]. The four-stage DEA is based on the results of the three-stage DEA model to construct a Tobit regression model to measure the impact of internal influences on the financing efficiency of a firm in a homogeneous environment. Therefore, this paper refers to the analytical structure of Lei and Liu [16] and adopts a four-stage DEA method to analyze the impact of external influences on the efficiency of green financing using the SFA model first, followed by the Tobit model to analyze the impact of internal influences on the efficiency of green financing.

### Construction of four-stage DEA model

**The first stage, the DEA-BCC model.** The input-oriented DEA-BCC model was chosen to measure the combined technical efficiency, pure technical efficiency and scale efficiency of listed companies in the construction industry. The model is shown in Eq (1):

$$min\theta -\varepsilon (\sum_{j=1}^{m}s_{j}^{-}+\sum_{r=1}^{n}s_{r}^{+})$$ (1)

Binding conditions:

$$\sum_{j=1}^{m}x_{ij}+s_{j}^{-}=\theta x_{0j},j=1,2,\ldots ,m$$

$$\sum_{r=1}^{s}\lambda_{i}x_{ir}-s_{r}^{+}=\theta y_{0r},r=1,2,\ldots ,s$$

$$\sum_{i=1}^{n}\lambda_{i}=1,\lambda_{i}\geq 0,i=1,2,\ldots ,n$$

where x_0j_ and y_0r_ represent the jth input and rth output of the ith firm, $s_{j}^{-}$ are slack variables for the jth input and $s_{r}^{+}$ are slack variables for the rth output. When θ = 0, s^−^ = 0, s^+^ = 0, then the decision unit is DEA efficient and technically optimal.

#### The second stage, the SFA model

This stage excludes the influence of external factors and random errors on inputs, and finally obtains the input redundancy of the decision unit caused by management inefficiency only. This model takes input slack variable as explained variable and external influencing factor as explanatory variable. The SFA regression model is shown in Eq (2):

$$S_{ij}=f(Z_{i},\beta_{j})+u_{ij}+v_{ij}$$ (2)

Where i = 1,2,…, n; j = 1,2,…, m; S_ij_ represents the jth input slack variable for the ith sample firm, f(Z_i_, β_j_) represents the effect of external influencing factors on the input slack variable, u_ij_ represents the management inefficiency term, v_ij_ represents the random error term, u_ij_ and is v_ij_ mutually independent. Using the formula of Akbarian [22] to estimate management inefficiency and calculate the adjusted input slack variables, as shown in Eq (3):

$$X_{ij}^{*}=X_{ij}+\{max[f(z_{i},\hat{\beta_{j}})]-f(z_{i},\hat{\beta_{j}})\}+[\max\left(v_{ij}\right)-v_{ij}]$$ (3)

Where i = 1,2,…, n; j = 1,2,…, m; $X_{ij}^{*}$ representing the jth input variable adjusted value for the i-th decision unit. $\left\{max[f(z_{i},\hat{\beta_{j}})]-f(z_{i},\hat{\beta_{j}})\right\}$ eliminating the input redundancy caused by differences in external influencing factors that [max(v_ij_)−v_ij_] so that all firms face the same business luck. The adjusted input redundancy reflects only the management inefficiency term.

In the third stage, the input variables are adjusted according to the SFA results and then the true green financing efficiency values are evaluated through the DEA-BCC model.

The adjusted values of the input variables and the initial values of the output variables obtained in the second stage are brought into the DEA-BCC model and the true green financing efficiency values of the decision units are again calculated.

#### The fourth stage, Tobit model

Based on the obtained modified green financing efficiency values of the sample companies as the explanatory variables and the internal influencing factors of each company as the explanatory variables. Build the Tobit model as shown in Eq (4)

$$Y_{i}=f(x_{ij},\beta_{ij})+u_{ij}$$ (4)

Where i = 1, 2, …, n; j = 1, 2, …, m; Y_i_ denotes the green financing efficiency value of the ith sample company; x_ij_ is the jth internal ecological factor of the ith sample company that affects its green financing efficiency value, and u_ij_ is a random error term.

### Data sources and selection

Considering that the data of listed companies are more readily available, this paper takes listed construction companies as the research object, determines the scope of listed construction companies according to the industry classification results of listed companies published by the Securities and Futures Commission from 2019–2020. The samples were screened according to the following conditions: (1) exclude samples with abnormal or missing financial data; (2) exclude samples whose main business is not in the scope of the construction industry; (3) exclude samples that are engaged in green building projects; (4) exclude samples that have the ST logo in the current year. "The Green Building Evaluation Standard" was released in 2019, standardizing the evaluation and recognition of green buildings. Therefore, 2019 was taken as the starting point for the research. Finally, 41 listed companies were selected as sample companies as shown in Table 1. The raw data for the subsequent research was obtained from the National Statistical Yearbook, public data from the People’s Bank of China, and the Tong Hua Shun iFinD.

**Table 1:** Sample companies.

Serial number	Company name	Serial number	Company name	Serial number	Company name
1	High-tech development	15	Pudong Construction	29	Hongtao shares
2	Chongqing Construction	16	Anhui Construction	30	Yaxia Corporation
3	Tianjian Group	17	Tengda Construction	31	Guangtian Group
4	Sinosteel International	18	Tongji Technology	32	Ruihe shares
5	CIGI	19	Long Jiang shares	33	Chisin Corporation
6	Guangdong Hydropower	20	China Railway Construction	34	China Decoration Construction
7	Southeastern Mesh Frame	21	China Central Railway	35	Jialu shares
8	Tung Wah Technology	22	China Zhongye	36	Famous Artists
9	Oriental Garden	23	China Construction	37	Hangxiao Steel Structure
10	Sinochem Geotechnical	24	CEC	38	River Group
11	Dove	25	China Communications Construction	39	Quanzhu
12	Arts & Gardens	26	Yuancheng Co.	40	Centurion shares
13	Xinjiang Jiaotong Construction	27	Baoying shares	41	Colida
14	Donghu Hi-Tech	28	Golden Mantis		

### Indicator construction

Under the guidance of green finance theory with Chinese characteristics and input-output efficiency theory, the indicator construction of this paper includes the selection of input-output indicators to measure the green financing efficiency of listed companies in the construction industry, as well as the screening of internal and external factors affecting the green financing efficiency. In the selection of input-output indicators, try to fit the development status of listed companies in China’s green finance and construction industry, and consider risk factors. In the screening of internal and external influencing factors, based on the fact that the real market environment is more complex than the ideal market, there are problems such as transaction friction, information asymmetry, and imperfect internal governance, which will inevitably hinder the green financing of listed companies in the construction industry, and the use of financing funds is not optimal, and ultimately affect the efficiency of green financing. Therefore, the factors that have an impact on the green financing efficiency of listed companies in the construction industry but are not within the subjective controllable range of the sample are selected as external influencing factors, and the factors that may have an impact on the green financing efficiency of listed companies in the construction industry but can be improved and changed through the company’s efforts are selected as internal influencing factors. The details are as follows:

Firstly, the selection of input-output indicators. Under the background of carbon neutral, this paper regards the listed construction companies with environmental factors as the financing target, through green financial instruments to obtain financing as green financing. Its use efficiency into green funds as green financing efficiency. Considering the characteristics of the main green capital sources of construction enterprises such as green credit, green bonds and green stocks, the selected indicators are required to meet the principles of effectiveness, comparability, applicability and availability. Finally, green debt financing cost, green equity financing capacity, green financing scale and green financing structure are selected as input indicators. Among them, the green debt financing cost was borrowed from Xie et al., in which the cost of green debt financing is the sum of current interest expenditure divided by interest-bearing liabilities. Interest-bearing liabilities include short-term loans, non-current liabilities due within one year, long-term loans and bonds payable [23]. Green equity financing capacity, green financing scale and green financing structure learn from the practices of Zhang et al., which are expressed by the proportion of institutional shareholding in the company’s outstanding shares, total assets and asset-liability ratio [18]. In terms of output indicators, drawing on the practice of most scholars, the return on net assets, earnings per share, total asset turnover, and operating income are used as indicators to measure corporate profitability and operating capacity. The specific evaluation indicators are shown in Table 2.

**Table 2:** Description of input-output indicators.

Category 1 indicators	Category 2 indicators	Measurement methods
Input indicators	Green debt financing costs	Interest expense/(short-term borrowings + non-current liabilities due within one year + long-term borrowings + bonds payable)
	Green equity financing capacity	Institutional holdings as a percentage of the company’s outstanding shares
	Scale of Green Financing	Total assets
	Green Financing Structure	Total liabilities/total assets
Output indicators	Return on net assets	Net profit/net assets
	Earnings per share	Profit after tax/net assets
	Total asset turnover ratio	Operating income/average total assets
	Operating income	Income from main business + other business income

Secondly, the selection of external influencing factors. The factors that affect the green financing efficiency of listed construction companies but are not within the subjective controllable range of the sample are selected as external influencing factors. Including industrial development environment, financial environment, legal environment, external factors are as follows:

1. Industry development support. Referring to the studies of Kim and Lee, Wu and Yin, Wang and Lu, the "Local Green Finance Development Index and Assessment Report" comprehensively measures the government’s policy support for green finance based on two aspects of policy promotion and market effect, and more comprehensively reflects the market effect of local green finance [24–26]. Therefore, this index is obtained from the "Local Green Finance Development Index and Assessment Report", which is published by the Green Finance International Research Institute of Central University of Finance and Economics, to measure the development environment of local green building industry.
2. Financial development level. Many scholars use indicators such as total bank credit as a share of GDP and total securities market financing as a share of GDP to measure the level of financial development. But these indicators do not highlight the total amount of total financial support received by the real economy from the financial system. Therefore, referring to the study of Liu and Sun, the level of financial development is measured by using the incremental social financing scale of each province, which is the total amount of funds obtained by the real economy through the financial system in that province in that year [27]. It can measure the total amount of funds obtained by the real economy from the financial sector in a more comprehensive way. Listed construction companies have more green financing channels and easier access to green funds in areas with high financial development levels.
3. The number of patent authorizations. Patent data are widely used in various types of research, due to the advantages of objectivity, reliability and easy accessibility, and contain rich technical information [28]. Granted patents indicate that the patents have the corresponding legal effect and their inventive value has been recognized by the review. And to a certain extent, they can represent the degree of legal protection of regional intellectual property rights. The effective protection of intellectual property rights of enterprises helps to improve their capital allocation efficiency.

Thirdly, the selection of internal influencing factors. Considering that the internal ecological factors of enterprises are mainly divided into two aspects, namely human resources and financial resources. The main measurement is the impact of six enterprise-level internal influencing factors on the green financing efficiency of listed companies in the construction industry, including nature of ownership, proportion of independent directors, ownership concentration, liability structure, proportion of R&D investment, and main business cost rate. Among them, ownership concentration is measured by the practice of Ilhan-Nas et al., through the shareholding ratio of the first largest shareholder; the larger the ratio, the higher the equity concentration of the sample enterprises, and vice versa, the lower it is [29]. The details are shown in the Table 3.

**Table 3:** Description of internal influencing factors.

Variables	Measurement methods
Nature of business ownership	State-owned enterprises take the value of 1 and non-state-owned take 0
Proportion of independent directors	Number of independent directors/ the total number of board of directors
Ownership concentration	Percentage of shareholding of the largest shareholder
Current liabilities ratio	Current liabilities/total liabilities
Proportion of R&D investment	R&D investment/operating income
Main business cost rate	Cost of main operations/income from main operations

## Analysis of empirical results

### Analysis on green financing efficiency of listed construction companies in the first stage

The first stage uses the DEA-BBC model and DEAP2.1 software to analyze the input-output data of 41 listed construction companies in China from 2019 to 2020. The results are shown in Table 4.

**Table 4:** Results of green financing efficiency of listed construction companies in the first stage.

Serial number	Company name	2019			2020		
		TE	PTE	SE	TE	PTE	SE
1	High-tech development	0.803	0.841	0.956	1	1	1
2	Chongqing Construction	0.572	0.746	0.767	0.597	0.645	0.927
3	Tianjian Group	1	1	1	1	1	1
4	Sinosteel International	0.683	0.685	0.997	0.755	0.779	0.969
5	CIGI	1	1	1	0.565	1	0.565
6	Guangdong Hydropower	0.465	0.743	0.625	0.384	0.624	0.615
7	Southeastern Mesh Frame	0.857	0.876	0.979	0.791	0.861	0.918
8	Tung Wah Technology	1	1	1	1	1	1
9	Oriental Garden	0.144	0.682	0.211	0.161	0.74	0.218
10	Sinochem Geotechnical	0.533	0.806	0.662	0.554	0.852	0.65
11	Dove	1	1	1	1	1	1
12	Arts & Gardens	1	1	1	0.966	1	0.966
13	Xinjiang Jiaotong Construction	0.867	1	0.867	0.816	1	0.816
14	Donghu Hi-Tech	0.376	0.801	0.47	0.891	0.924	0.964
15	Pudong Construction	0.623	0.828	0.753	0.876	0.967	0.906
16	Anhui Construction	0.729	0.873	0.835	0.59	0.681	0.866
17	Tengda Construction	0.72	0.934	0.771	1	1	1
18	Tongji Technology	1	1	1	1	1	1
19	Long Jiang shares	0.754	0.823	0.916	0.586	0.756	0.775
20	China Railway Construction	1	1	1	1	1	1
21	China Central Railway	1	1	1	1	1	1
22	China Zhongye	0.783	0.838	0.933	0.775	0.814	0.952
23	China Construction	1	1	1	1	1	1
24	CEC	0.594	0.819	0.725	1	1	1
25	China Communications Construction	0.945	0.985	0.96	0.675	0.894	0.755
26	Yuancheng Co.	1	1	1	1	1	1
27	Baoying shares	0.666	0.814	0.818	0.479	0.803	0.596
28	Golden Mantis	1	1	1	1	1	1
29	Hongtao shares	0.348	0.82	0.425	0.267	0.723	0.369
30	Yaxia Corporation	0.524	0.851	0.616	0.515	0.845	0.609
31	Guangtian Group	0.462	0.64	0.722	0.406	0.71	0.572
32	Ruihe shares	0.825	0.875	0.943	0.891	0.995	0.896
33	Chisin Corporation	1	1	1	0.995	1	0.995
34	China Decoration Construction	1	1	1	1	1	1
35	Jialu shares	0.581	0.713	0.815	0.529	0.74	0.715
36	Famous Artists	0.677	1	0.677	0.367	1	0.367
37	Hangxiao Steel Structure	1	1	1	1	1	1
38	River Group	0.549	0.69	0.796	0.938	0.966	0.971
39	Quanzhu	0.725	0.771	0.941	0.523	0.705	0.743
40	Centurion shares	1	1	1	1	1	1
41	Colida	0.829	0.944	0.878	0.927	0.987	0.939
	mean	0.772	0.888	0.855	0.776	0.903	0.845

The calculation results of the DEA-BCC model show that:

1. From the overall perspective of the sample, the average green financing efficiency of listed construction companies in 2019 and 2020 are 0.772 and 0.776 respectively, indicating that the green financing efficiency of listed construction companies is not high and and the green advantage is not large. Among them, the average value of pure technical efficiency is 0.888 and 0.903 respectively, the average value of scale efficiency is 0.855 and 0.845 respectively. The average value of pure technical efficiency is more than 0.9 in 2020, which is at a high level. It shows that the listed companies in the construction industry mainly rely on the regulation of market factors and the improvement of management level to improve the efficiency of green financing and have achieved certain results. Efficiency loss mainly comes from poor scale efficiency.
2. In the sample, 36.59% and 34.15% of the listed construction companies in 2019 and 2020 respectively achieved strong DEA efficiency, that is, each efficiency value is 1. 41.46% and 46.34% were relatively effective in pure technology; 36.59% and 34.15% are relatively effective at achieving scale efficiency. A large number of listed construction companies need to improve both pure technical efficiency and scale efficiency. Scale efficiency is the main factor restricting green financing efficiency.
3. In the samples of 2019 and 2020, 60.98% and 63.41% of the listed companies in the construction industry are in the stage of increasing returns to scale, indicating that more than half of the listed companies in the construction industry need to expand their scale.

Because the results include the external influencing factors and random factors, it cannot reflect the real green financing efficiency of listed construction companies, so further adjustment and calculation are needed.

### Analysis of the impact of external factors on green financing efficiency in the second stage

Taking the input slack variables obtained in the first stage as the dependent variables, the three external influencing factors defined before as the independent variables, the SFA regression model is constructed by using software Frontier4.1. According to the SFA calculation results, the influence of external influencing factors and random errors on the redundancy of each input variable is analyzed, and the input variables are adjusted according to the results. The results of the regression analysis are shown in Tables 5 and 6.

**Table 5:** SFA estimation results of input slack variables of listed construction companies in the second stage of 2019.

	Green debt financing costs	Green equity financing capacity	Scale of Green Financing	Green Financing Structure
Intercept term	-7.5823345***	-42.764106***	-2549.9794***	2.9828932***
Industry Development Support	-2.4613313**	-21.812364***	-285.51025***	10.010333***
Level of financial development	9.4644162***	56.062***	3079.1387***	-8.2613768***
Number of patents granted	-5.3865409***	-30.009405***	-1946.8143***	2.3266545**
sigma-squared	10.403564***	112.09203***	972453.16***	187.94786***
gamma	0.999999***	0.999999***	0.999999***	0.999999***
log likelihood	-60.045207	-128.02417	-306.79702	-139.82909
LR	32.144831***	15.646247***	31.287894***	13.53539***

***, ** and * represent significant at the 1%, 5% and 10% levels respectively.

**Table 6:** SFA estimation results of input slack variables of listed construction companies in the second stage of 2020.

	Green debt financing costs	Green equity financing capacity	Scale of Green Financing	Green Financing Structure
Intercept term	0.69658459	-7.7168892***	-484.02521***	-7.77602***
Industry Development Support	2.022907**	-7.5370758***	2230.1905***	9.1276869***
Financial development level	-3.6317837**	9.8691569***	-1004.107***	-5.3042836***
The number of patent authorizations	1.7834928*	-4.2783923***	152.95566***	2.7091681**
sigma-squared	6.5869626***	565.82319***	1124528***	216.36043***
gamma	-	0.999999***	0.999999***	0.999999***
log likelihood	-72.595477	-148.22653	-306.19233	-133.33397
LR	-	41.497794******	39.089328***	31.388136***

***, ** and * represent significant at the 1%, 5% and 10% levels respectively.

The LR one-sided error, sigma-squared and gamma of the empirical results have basically passed the significance test. The estimated results of the model are generally acceptable, and most of the estimated values of each parameter are significant. Among them, because the 2020 green debt financing cost did not pass the LR test, it is not discussed.

It is verified that there is a relationship between external influencing factors and input redundancy of listed companies in the construction industry. It should be noted that since the external influencing factors are the regression of each input slack variable, when the regression coefficient is negative, it means that increasing the value of external influencing factors is conducive to reducing the input slack of listed construction companies, which is conducive to reducing waste and bringing positive effects. When the regression coefficient is positive, it means that increasing external factors will increase the investment slack of listed construction companies, that is, increase waste and bring negative effects.

According to the SFA regression coefficient of the external influencing factors and input slack variables, the following conclusions can be drawn:

1. Industry development support, the influence coefficient on the green equity financing capacity slack has been significantly negative at the level of 1%, the influence coefficient on the green financing scale slack has changed from negative to positive and passed the significance test of 1%, and the influence coefficient on the green financing structure slack has always been significantly positive at the level of 1%. This shows that the increase of industrial development support, that is, the increase of green financial support, will attract institutional investors to scientifically hold shares in listed construction companies, while the listed construction companies will have irrational financing decisions because of policy support, integrate too much green funds, and have structural risks.
2. Financial development level, the influence coefficient on the green equity financing capacity slack is always positive and passes the 1% significance test. The influence coefficient on the green financing scale slack turns from positive to negative and is significant at the 1% level. The influence coefficient on the green financing structure slack is always significantly negative at the 1% level. This shows that the improvement of financial development level has not brought favorable institutional investors to listed construction companies. At this time, the listed construction companies will also make more rational decisions to control the scale of financing, and the risk of financing structure is small.
3. The number of patent authorizations, the coefficient of influence on the green equity financing capacity slack has always remained significant at the 1% level. The coefficient of influence on the green financing scale slack has changed from negative to positive and significant at the 1% level. The coefficient of influence on the green financing structure slack is always positive and passes the 5% significance level test. This shows that the increase in the number of patent authorizations, that is, the attention of the society to intellectual property rights can promote listed construction companies to develop their own intellectual property rights and attract institutional investors to invest scientifically. But there is also a phenomenon of excessive financing scale and structural risks.

On the whole, external influencing factors will have a complex impact on listed construction companies. Listed construction companies need to dialectically treat changes in the external environment.

### Analysis on green financing efficiency of listed construction companies in the third stage of homogeneous environment

In the third stage, use the adjusted input and original output data to re-estimate the real green financing efficiency of listed construction companies. The differences of TE, PTE and SE between the third stage and the first stage are compared and analyzed. The results are shown in Table 7.

**Table 7:** Results and comparison of green financing efficiency of listed construction companies in the third stage.

	2019 The first stage	2019 The third stage	2020 The first stage	2020 The third stage
TE	0.772	0.710	0.776	0.748
PTE	0.888	0.790	0.903	0.900
SE	0.855	0.880	0.845	0.818

1. In 2019, the technical efficiency and pure technical efficiency of listed construction companies in the third stage decreased, while the scale efficiency increased. Compared with the results of the third stage, the technical efficiency loss caused by pure technical inefficiency obtained in the first stage is underestimated, and the technical efficiency loss caused by scale inefficiency is overestimated.
2. In 2020, the technical efficiency, pure technical efficiency and scale efficiency of listed construction companies in the third stage all decline. Compared with the results of the third stage, the first stage obtains that the technical efficiency loss caused by the scale inefficiency is underestimated.
3. In the third stage, 25 listed construction companies are in the stage of increasing returns to scale, accounting for 60.98% of the sample, indicating that more than half of the listed construction companies need to expand their scale.

### Analysis of the impact of internal factors on green financing efficiency in the fourth stage

In the fourth stage, the technical efficiency of listed construction companies in a homogeneous environment obtained in the third stage of DEA were chosen as the explanatory variables. Based on Tobit model, the influence of internal financing environment variables on green financing efficiency is calculated by using STATA14.0

As shown in Table 8. The nature of business ownership is negatively correlated with green financing efficiency, but it does not pass the significance test; ownership concentration, current liabilities ratio, and the main business cost rate are positively correlated with green financing efficiency, but they have not passed the significance test.

**Table 8:** Tobit regression results of the impact of internal factors on green financing efficiency in the fourth stage.

Variables	Coef.	t	P>|t|
Nature of business ownership	-0.03106	-0.42	0.673
Proportion of independent directors	0.7143823	2.01	0.048**
Ownership concentration	0.2717864	1.05	0.299
Current liabilities ratio	0.1741187	0.72	0.474
Proportion of R&D investment	-3.70777	-1.72	0.09*
Main business cost rate	0.0531481	0.13	0.893

***, ** and * represent significant at the 1%, 5% and 10% levels respectively.

The proportion of independent directors and the proportion of R&D investment pass the significance test, so the analysis is carried out around these two internal influencing factors.

The proportion of independent directors is positively correlated with the efficiency of green financing and passes the 5% significance test. This shows that the better the independence of the board of directors is, the more conducive it is to supervise the management and promote the rational decision-making of listed construction companies, finally improving green financing efficiency.

The proportion of R&D investment is negatively correlated with green financing efficiency and passes the 10% significance test, which shows that increasing the proportion of R&D investment does not bring positive effects. It may have a negative impact on the green financing efficiency of listed construction companies due to the low conversion rate of achievements and the mismatch between R&D input and output.

## Research conclusions and implications

### Research conclusions

Based on the four-stage DEA model calculation and analysis, this paper introduces the green finance theory with Chinese characteristics and input-output efficiency theory at the theoretical level, uses the idea of input-output analysis to evaluate the current situation of green financing efficiency of listed companies in the construction industry in the context of Chinese characteristics, and studies the impact of internal and external factors on green financing efficiency. So as to further expand the research cases of green finance theory system with Chinese characteristics and provide experience reference and ideas for other researches on the development of green industry.

The results show that:

1. Under homogeneous environmental conditions, the current green financing efficiency of listed construction companies is still at a low level.
2. External influencing factors and random factors will have a significant complex impact on the green financing efficiency of listed construction companies, they need to be treated dialectically.
3. More than 60% of the listed construction companies are still in the stage of increasing returns to scale. Green financing needs of many listed construction companies are not met, need to improve the intensity of green finance.
4. The increase in the proportion of independent directors can effectively increase the green financing efficiency of listed construction companies, while in the case of low efficiency of capital interests of listed construction companies, the increase in R&D investment will have a negative effect, reducing the green financing efficiency.

### Suggestions for countermeasures

In view of the low efficiency of green financing of listed companies in China’s construction industry to effectively improve it, the following suggestions are put forward in conjunction with the current situation of green finance development and the findings of the experimental study:

1. Listed construction companies need expand their scale reasonably and the government should give green financial support. The research results show that a large proportion of listed construction companies are still in the stage of incremental scale compensation, and their green financing needs have not been met. To achieve scale operation, they still need to reasonably expand their green financing scale, which requires enhancing the support of green finance to the green development of the construction industry and developing more green financial products to meet the needs.
2. Improving the external environment of green financing of listed construction companies. The government should create positive external conditions for listed construction companies to effectively improve green financing efficiency and play its policy guiding role. The government implements targeted policies based on the overall low green financing efficiency of listed construction companies and the characteristics of external factors such as local industrial support, financial development level, and number of patent authorizations.
3. Optimize the internal management environment of green financing of listed construction companies. Listed construction companies need to strengthen their own internal organization, resource allocation structure and management, so that the funds in the existing enterprise technology management level can be fully utilized to maximize economic efficiency. According to the Tobit regression results, listed construction companies should increase the number of independent directors in the management to improve the independence of the board of directors, while paying attention to the benefits of R&D investment and avoiding low-quality R&D investment.

## Supporting information

S1 Appendix Sample enterprise data. (PDF)

## References

[1] China Building Energy Consumption Research Report 2020. Building Energy Efficiency (Chinese and English). 2021;02:1–6.

[2] Fan YP, Zhou J. Green finance theory with Chinese characteristics under the "double carbon" goal: historical mirror and practice direction. Economic Issues. 2022;09:1–8. doi: 10.16011/j.cnki.jjwt.2022.09.002

[3] Wang W, Tian Z, Xi WJ, Tan YR, Deng Y. The influencing factors of China’s green building development: An analysis using RBF-WINGS method. Building and Environment. 2020;188:107425. 10.1016/j.buildenv.2020.107425

[4] Salazar Jose., Environmental Finance: Linking Two Worlds. Bratislava: Slovakia; 1998.

[5] Ma J. Outlook for green finance in China. China Finance. 2016;16:20–22.

[6] Ning YY, Cherian J, Sial MS, Alvarez-Otero S, Comite U, Zia-Ud-Din M. Green bond as a new determinant of sustainable green financing, energy efficiency investment, and economic growth: a global perspective. Environmental Science and Pollution Research. 2022;06. https://doi.org/1007/s11356-021-18454-7 10.1007/s11356-021-18454-7 PMC8735735 34993809

[7] Zeng G, Guo HX, Geng CX. Mechanism analysis of influencing factors on financing efficiency of strategic emerging industries under the "dual carbon" background: evidence from China. Environmental Science and Pollution Research.2022;09. doi: 10.1007/s11356-022-22820-4 36064853 PMC9444705

[8] Lian YH, Gao JY, Ye T. How does green credit affect the financial performance of commercial banks?—Evidence from China. Journal of Cleaner Production. 2022;344:131069. 10.1016/j.jclepro.2022.131069

[9] Liu S, Xu RX, Chen XY. Does green credit affect the green innovation performance of high-polluting and energy-intensive enterprises? Evidence from a quasi-natural experiment. Environmental Science and Pollution Research. 2021;28:65265–65277. doi: 10.1007/s11356-021-15217-2 34231142

[10] Zhang L, Xiao L, Gao JF. Measurement and comparison of the level and efficiency of green finance development in China—micro data based on 1040home public companies. China Science and Technology Forum. 2018;9:100–112+120. doi: 10.13580/j.cnki.fstc.2018.09.017

[11] Yu YG, Yan YN, Shen PY, Li YT, Ni TH. Green Financing Efficiency and Influencing Factors of Chinese Listed Construction Companies against the Background of Carbon Neutralization: A Study Based on Three-Stage DEA and System GMM. Axioms. 2022;11:467. 10.3390/axioms11090467

[12] Lee CC, Lee CC. How does green finance affect green total factor productivity? Evidence from China. Energy Economic. 2022;107:105863. 10.1016/j.eneco.2022.105863

[13] Lin JD, Chen JL, Qiu GY. Research on green financial support factors of China’s environmental protection industry—an empirical analysis based on CSI environmental protection industry 50index constituents. Industrial Technology Economics. 2018;37:129–135.

[14] Cao TQ, Zhang CY, Yang X. Green effects and impact mechanisms of green credit policies—Evidence based on green patent data of Chinese listed companies. Financial Forum. 2021;26:7–17. doi: 10.16529/j.cnki.11-4613/f.2021.05.003

[15] Qiao Q, Fan J, Sun Y, Song QH. "Research on green finance measurement and influencing factors in provinces along the Belt and Road. Industrial Technology Economy. 2021;40:120–126.

[16] Lei H, Liu QY. Research on the financing efficiency of green low-carbon enterprises based on four-stage DEA model. Finance Theory and Practice. 2020;41:72–78. doi: 10.16339/j.cnki.hdxbcjb.2020.03.010

[17] Jin Y, Gao X, Wang M. The financing efficiency of listed energy conservation and environmental protection firms: Evidence and implications for green finance in China. Energy Policy. 2021;153:112254. 10.1016/j.enpol.2021.112254

[18] Zhang M, Zhang XL, Song Y, Zhu J. Exploring the impact of green credit policies on corporate financing costs based on the data of Chinese A-share listed companies from 2008 to 2019. Journal of Cleaner Production. 2022;375:134012. 10.1016/j.jclepro.2022.134012

[19] Ali AL, Lerme CS. Comparative advantage and disadvantage in DEA. Annals of Operations Research. 1997;73:215–232. 10.1023/A:1018929228294

[20] Panwar A, Olfati M, Pant M, Snasel V. A Review on the 40 Years of Existence of Data Envelopment Analysis Models: Historic Development and Current Trends. Archives of Computational Methods in Engineering. 2022;29:5397–5426. doi: 10.1007/s11831-022-09770-3 35702633 PMC9184254

[21] Fried H, Lovell C, Schmidt S, et al. Accounting for Environmental Effects and Statistical Noise in Data Envelopment Analysis. Journal of Productivity Analysis. 2002;17:121–136. doi: 10.1023/A:1013548723393 Corpus ID: 14358494

[22] Akbarian D. Network DEA based on DEA-ratio. Financial Innovation. 2021;7:73. 10.1186/s40854-021-00278-6

[23] Xie HB, Ding LF, Liao K. Overseas background directors and the cost of debt financing—mediating effects based on the advisory and supervisory functions of the board of directors. Management Review. 2019;31:202–211. doi: 10.14120/j.cnki.cn11-5057/f.2019.11.017

[24] Kim KH, Lee YH. Analyzing Relationship of Business Support Service Quality of Local Industrial Development Policy Influencing Enhanced Industrial Capabilities and Reuse Intention. Korean Policy Sciences Review. 2015;19:85–106. UCI: G704-000863.2015.19.4.007

[25] Wu HY, Yin DS. The "reward" and "penalty" of green credit policy on corporate debt financing: an assessment of the effect based on quasi-natural experiments. Contemporary Finance. 2021;435:49–62. doi: 10.13676/j.cnki.cn36-1030/f.2021.02.006

[26] Wang ZJ, Lu ZJ. Green finance, analysts’ concerns and new energy enterprises’ financing relief. Contemporary Finance and Economics. 2022;454:52–63. doi: 10.13676/j.cnki.cn36-1030/f.2022.09.005

[27] Liu XR, Sun T. Dynamic economic growth threshold effect of financial development on energy consumption in China. Contemporary Finance and Economics. 2019;08:48–57. doi: 10.13676/j.cnki.cn36-1030/f.2019.08.006

[28] Shinkle GA, Suchard JA. Innovation in newly public firms: The influence of government grants, venture capital, and private equity. Australian Journal of Management. 2019;44:248–281. 10.1177/0312896218802611

[29] Ilhan-Nas T, Okan T, Tatoglu E, Demirbag M, Glaister KW. The effects of ownership concentration and institutional distance on the foreign entry ownership strategy of Turkish MNEs. Journal of Business Research. 2018;93:173–183. 10.1016/j.jbusres.2018.02.006

