# Supplementary material for: Construction enterprises’ green financing efficiency and its influencing factors including internal and external: Based on four-stage DEA model
Source: PLoS One. 2023 Jun 8;18(6):e0286043. doi: 10.1371/journal.pone.0286043 (PMC10249804; doi:10.1371/journal.pone.0286043)
Supplement: S1 Appendix — (PDF) [file pone.0286043.s001.pdf]

Serial number	Year	Code	Company name	Province	Shareholding ratio	Asset
1	2019	000628	High-tech development	Sichuan	15.67%	5,881,496,980.76
2	2019	600939	Chongqing Construction	Chongqing	52.09%	71,844,640,260.56
3	2019	000090	Tianjian Group	Guangdong	55.03%	40,313,371,788.43
4	2019	000928	Sinosteel International	Jilin	41.21%	18,326,440,169.53
5	2019	002051	CIGI	Beijing	64.41%	21,999,483,304.15
6	2019	002060	Guangdong Hydropower	Guangdong	38.20%	26,047,764,064.96
7	2019	002135	Southeastern Mesh [illegible]	Zhejiang	60.52%	11,443,472,566.34
8	2019	002140	Tung Wah Technology	Anhui	65.96%	6,673,569,147.39
9	2019	002310	Oriental Garden	Beijing	29.23%	43,811,501,216.44
10	2019	002542	Sinochem Geotechnical	Beijing	46.90%	9,008,290,719.83
11	2019	002761	Dove	Zhejiang	67.33%	79,323,965,003.99
12	2019	002775	Arts & Gardens	Guangdong	38.26%	4,609,391,665.76
13	2019	002941	Xinjiang Jiaotong [illegible]	Xinjiang	25.56%	10,135,125,287.91
14	2019	600133	Donghu Hi-Tech	Hubei	34.41%	26,643,893,730.67
15	2019	600284	Pudong Construction	Shanghai	38.46%	14,484,707,880.46
16	2019	600502	Anhui Construction	Anhui	10.55%	90,435,503,647.10
17	2019	600512	Tengda Construction	Zhejiang	8.86%	10,244,697,333.01
18	2019	600846	Tongji Technology	Shanghai	26.55%	12,152,120,301.84
19	2019	600853	Long Jiang shares	Heilongjiang	33.91%	19,743,796,397.02
20	2019	601186	China Railway [illegible]	Beijing	79.93%	1,081,239,213,000.00
21	2019	601390	China Central Railway	Beijing	18.92%	352,997,821,000.00
22	2019	601618	China Zhongye	Beijing	75.35%	458,506,213,000.00
23	2019	601668	China Construction	Beijing	77.36%	2,034,451,929,000.00
24	2019	601669	CEC	Beijing	68.75%	813,227,622,329.43
25	2019	601800	China Communications [illegible]	Beijing	90.25%	1,120,399,492,938.00
26	2019	603388	Yuancheng Co.	Zhejiang	8.73%	2,937,720,624.41
27	2019	002047	Baoying shares	Guangdong	24.30%	10,208,933,593.72
28	2019	002081	Golden Mantis	Jiangsu	63.43%	39,473,391,654.97
29	2019	002325	Hongtao shares	Guangdong	9.11%	11,689,735,162.81
30	2019	002375	Yaxia Corporation	Zhejiang	40.17%	21,630,126,543.08
31	2019	002482	Guangtian Group	Guangdong	60.80%	24,444,193,026.03
32	2019	002620	Ruihe shares	Guangdong	32.91%	5,864,510,237.10
33	2019	002781	Chisin Corporation	Guangdong	50.65%	4,774,441,152.08
34	2019	002822	China Decoration [illegible]	Guangdong	5.00%	6,065,098,464.61
35	2019	300117	Jialu shares	Beijing	42.31%	6,098,650,790.55
36	2019	300506	Famous Artists	Guangdong	34.32%	4,073,628,639.21
37	2019	600477	Hangxiao Steel Structure	Zhejiang	3.13%	8,782,703,984.22
38	2019	601886	River Group	Beijing	47.40%	28,634,212,470.23
39	2019	603030	Quanzhu	Shanghai	9.51%	9,674,809,587.80
40	2019	603098	Centurion shares	Beijing	38.38%	4,708,805,840.99
41	2019	603828	Colida	Jiangsu	46.64%	3,993,201,484.49
42	2020	000628	High-tech development	Sichuan	16.03%	8,338,949,498.00
43	2020	600939	Chongqing Construction	Chongqing	87.64%	73,546,637,815.19
44	2020	000090	Tianjian Group	Guangdong	50.07%	48,998,633,233.39

45	2020	000928	Sinosteel International	Jilin	58.66%	19,708,894,841.57
46	2020	002051	CIGI	Beijing	64.50%	21,682,851,389.56
47	2020	002060	Guangdong Hydropower	Guangdong	37.75%	29,491,657,758.56
48	2020	002135	Southeastern Mesh [illegible]	Zhejiang	45.62%	13,650,090,612.56
49	2020	002140	Tung Wah Technology	Anhui	65.31%	8,134,712,984.23
50	2020	002310	Oriental Garden	Beijing	16.86%	45,379,124,099.99
51	2020	002542	Sinochem Geotechnical	Beijing	40.50%	10,603,917,630.56
52	2020	002761	Dove	Zhejiang	52.62%	86,780,759,638.82
53	2020	002775	Arts & Gardens	Guangdong	35.05%	5,394,068,605.48
54	2020	002941	Xinjiang Jiaotong [illegible]	Xinjiang	16.86%	14,607,666,697.17
55	2020	600133	Donghu Hi-Tech	Hubei	34.22%	27,178,372,195.78
56	2020	600284	Pudong Construction	Shanghai	43.35%	16,683,451,785.23
57	2020	600502	Anhui Construction	Anhui	51.38%	105,806,669,990.16
58	2020	600512	Tengda Construction	Zhejiang	1.65%	10,925,433,578.67
59	2020	600846	Tongji Technology	Shanghai	30.04%	11,318,133,979.77
60	2020	600853	Long Jiang shares	Heilongjiang	33.70%	23,446,262,778.74
61	2020	601186	China Railway [illegible]	Beijing	75.28%	1,242,792,799,000.00
62	2020	601390	China Central Railway	Beijing	14.70%	430,229,360,000.00
63	2020	601618	China Zhongye	Beijing	75.09%	506,392,963,000.00
64	2020	601668	China Construction	Beijing	71.64%	2,192,173,839,000.00
65	2020	601669	CEC	Beijing	63.05%	886,543,441,671.41
66	2020	601800	China Communications [illegible]	Beijing	86.61%	1,304,168,590,053.00
67	2020	603388	Yuancheng Co.	Zhejiang	13.65%	3,027,866,277.67
68	2020	002047	Baoying shares	Guangdong	39.05%	12,028,846,647.65
69	2020	002081	Golden Mantis	Jiangsu	65.57%	45,003,313,428.27
70	2020	002325	Hongtao shares	Guangdong	8.82%	12,311,599,111.23
71	2020	002375	Yaxia Corporation	Zhejiang	46.67%	22,280,486,748.03
72	2020	002482	Guangtian Group	Guangdong	56.87%	23,410,737,751.72
73	2020	002620	Ruihe shares	Guangdong	31.34%	6,354,774,421.15
74	2020	002781	Chisin Corporation	Guangdong	55.90%	3,818,140,537.35
75	2020	002822	China Decoration [illegible]	Guangdong	1.84%	7,302,375,657.32
76	2020	300117	Jialu shares	Beijing	41.89%	5,513,566,072.65
77	2020	300506	Famous Artists	Guangdong	18.94%	3,056,173,207.35
78	2020	600477	Hangxiao Steel Structure	Zhejiang	2.03%	8,964,703,841.12
79	2020	601886	River Group	Beijing	47.09%	29,381,850,084.19
80	2020	603030	Quanzhu	Shanghai	7.66%	10,508,589,843.16
81	2020	603098	Centurion shares	Beijing	33.34%	5,014,554,912.99
82	2020	603828	Colida	Jiangsu	44.63%	5,272,150,850.23

Asset-liability ratio	ROE	EPS	Operating revenue	Government support	Financial development level	Patent authorizations	Property nature	Ratio of independent
81.21%	11.09%	0.3360	3,310,871,524.72	58.940	9,653	82,066	1	33.33%
85.52%	6.21%	0.2300	52,108,517,978.16	38.440	5,970	43,872	1	37.50%
76.04%	13.42%	0.6042	14,665,288,591.27	73.110	29,190	527,390	1	42.86%
71.53%	10.85%	0.4255	13,414,075,951.86	10.000	2,965	15,579	1	42.86%
49.93%	10.14%	0.8500	10,656,800,243.72	56.710	14,630	131,716	1	50.00%
86.93%	7.48%	0.1946	11,143,485,046.29	73.110	29,190	527,390	1	36.36%
61.49%	6.44%	0.2600	8,976,374,629.37	68.370	22,162	285,342	0	33.33%
66.28%	8.16%	0.3300	4,517,995,996.30	35.710	7,255	82,524	1	42.86%
71.04%	0.41%	0.0200	8,133,197,164.62	56.710	14,630	131,716	1	33.33%
53.96%	6.30%	0.1400	4,100,278,615.93	56.710	14,630	131,716	1	33.33%
92.46%	16.25%	0.8500	75,649,475,026.19	68.370	22,162	285,342	1	42.86%
43.30%	9.68%	0.4780	2,898,628,461.74	73.110	29,190	527,390	0	33.33%
75.38%	8.06%	0.2900	5,951,176,397.62	44.690	2,948	8,652	1	37.50%
77.70%	4.03%	0.2193	9,423,207,629.11	33.420	8,734	73,940	1	37.50%
56.31%	6.84%	0.4208	6,219,610,574.93	29.130	8,642	100,587	1	33.33%
84.15%	6.53%	0.3500	47,265,560,036.00	35.710	7,255	82,524	1	33.33%
55.15%	10.64%	0.3000	4,367,956,951.29	68.370	22,162	285,342	0	42.86%
70.60%	23.95%	0.9700	6,351,036,402.54	29.130	8,642	100,587	1	42.86%
88.86%	13.35%	0.2665	11,106,371,251.76	17.290	3,097	19,989	1	40.00%
75.77%	12.03%	1.4000	830,452,157,000.00	56.710	14,630	131,716	1	11.11%
76.76%	12.84%	0.9500	848,440,346,000.00	56.710	14,630	131,716	1	50.00%
74.50%	7.99%	0.2700	338,637,609,000.00	56.710	14,630	131,716	1	57.14%
75.33%	15.60%	0.9700	1,419,836,588,000.00	56.710	14,630	131,716	1	66.67%
76.21%	8.45%	0.4567	347,712,701,074.05	56.710	14,630	131,716	1	33.33%
73.55%	10.22%	1.1600	554,792,365,252.00	56.710	14,630	131,716	1	28.57%
63.45%	14.20%	0.4800	1,007,890,013.01	68.370	22,162	285,342	0	33.33%
59.48%	5.14%	0.1600	6,676,833,260.14	73.110	29,190	527,390	1	42.86%
60.99%	16.55%	0.8800	30,834,654,530.30	54.970	24,104	314,395	0	37.50%
68.43%	1.20%	0.0292	4,030,647,812.96	73.110	29,190	527,390	0	42.86%
61.77%	5.40%	0.3200	10,785,629,767.25	68.370	22,162	285,342	0	50.00%
71.13%	2.08%	0.0900	13,046,256,284.52	73.110	29,190	527,390	0	37.50%
55.67%	6.98%	0.4300	3,817,998,179.36	73.110	29,190	527,390	0	33.33%
57.62%	4.42%	0.3800	4,014,476,168.05	73.110	29,190	527,390	1	37.50%
52.38%	10.81%	0.4100	4,859,107,862.49	73.110	29,190	527,390	0	42.86%
77.21%	1.85%	0.0400	3,437,198,147.11	56.710	14,630	131,716	0	42.86%
47.68%	7.60%	0.2300	1,252,031,928.05	73.110	29,190	527,390	0	33.33%
58.21%	14.31%	0.2180	6,633,068,516.72	68.370	22,162	285,342	0	42.86%
68.17%	4.70%	0.3100	18,805,181,163.52	56.710	14,630	131,716	0	33.33%
74.37%	10.59%	0.3900	6,936,116,180.23	29.130	8,642	100,587	0	37.50%
56.07%	11.06%	0.4400	3,356,560,906.93	56.710	14,630	131,716	0	58.33%
70.62%	3.32%	0.0700	2,287,030,202.21	54.970	24,104	314,395	0	33.33%
79.73%	20.73%	0.7650	5,533,078,836.61	46.130	14,334	82,066	1	33.33%
86.55%	3.08%	0.0900	55,294,166,859.98	29.730	8,101	55,377	1	37.50%
77.47%	14.31%	0.7380	17,124,707,926.40	56.560	40,692	709,725	1	50.00%

70.79%	11.19%	0.4790	14,827,488,855.31	17.720	3,579	23,951	1	37.50%
50.68%	-7.11%	-0.0700	7,965,990,108.72	57.100	16,631	162,824	1	42.86%
86.97%	8.04%	0.2192	12,583,024,873.89	56.560	40,692	709,725	1	40.00%
64.44%	6.24%	0.2600	9,256,289,931.66	57.040	32,155	391,700	0	33.33%
70.45%	8.65%	0.3701	5,210,304,765.89	33.920	9,251	119,696	1	50.00%
70.71%	-4.04%	-0.1800	8,725,535,437.62	57.100	16,631	162,824	1	33.33%
61.33%	4.57%	0.1000	5,662,781,314.71	57.100	16,631	162,824	1	33.33%
91.54%	24.73%	0.9500	79,549,653,110.03	57.040	32,155	391,700	1	42.86%
53.13%	6.29%	0.3100	2,520,203,789.55	56.560	40,692	709,725	0	33.33%
73.68%	4.65%	0.1800	7,139,197,770.34	34.420	4,558	12,763	1	37.50%
74.04%	13.65%	0.8700	10,593,750,640.33	30.650	10,433	110,102	1	37.50%
60.18%	7.09%	0.4582	8,376,485,796.62	31.990	10,916	139,780	1	33.33%
83.61%	9.01%	0.4700	56,972,444,417.50	33.920	9,251	119,696	1	33.33%
52.68%	12.18%	0.3800	5,903,418,678.10	57.040	32,155	391,700	0	42.86%
70.60%	20.09%	0.9500	6,302,631,317.57	31.990	10,916	139,780	1	42.86%
85.86%	11.81%	0.2234	11,837,428,005.59	22.250	1,899	28,475	1	36.36%
74.76%	11.45%	1.5000	910,324,763,000.00	57.100	16,631	162,824	1	50.00%
73.90%	11.85%	0.9630	971,404,889.00	57.100	16,631	162,824	1	42.86%
72.28%	8.93%	0.3200	400,114,623,000.00	57.100	16,631	162,824	1	60.00%
73.67%	15.54%	1.0700	1,615,023,327,000.00	57.100	16,631	162,824	1	57.14%
74.74%	7.85%	0.4632	401,180,654,966.12	57.100	16,631	162,824	1	33.33%
72.56%	7.12%	0.9000	627,586,194,472.00	57.100	16,631	162,824	1	42.86%
62.87%	894.00%	0.3200	714,763,820.37	57.040	32,155	391,700	0	37.50%
64.93%	2.68%	0.0800	5,954,903,194.19	56.560	40,692	709,725	1	33.33%
61.90%	14.83%	0.8800	31,243,227,802.04	46.650	33,611	499,167	0	33.33%
72.65%	-11.86%	-0.2797	3,568,577,321.30	56.560	40,692	709,725	0	42.86%
61.49%	3.89%	0.2400	10,787,352,160.12	57.040	32,155	391,700	0	33.33%
72.83%	-12.00%	-0.5100	12,246,478,061.74	56.560	40,692	709,725	0	33.33%
55.87%	6.15%	0.4000	3,763,882,372.44	56.560	40,692	709,725	0	33.33%
62.65%	-32.49%	-2.4700	2,109,572,607.08	56.560	40,692	709,725	1	33.33%
52.67%	8.32%	0.3700	5,581,448,926.63	56.560	40,692	709,725	0	42.86%
73.57%	5.66%	0.1100	2,078,633,676.06	57.100	16,631	162,824	0	33.33%
52.22%	-23.02%	-0.5300	512,877,750.71	56.560	40,692	709,725	0	33.33%
52.68%	19.42%	0.3360	8,138,757,441.89	57.040	32,155	391,700	0	42.86%
66.02%	11.55%	0.8400	18,049,964,771.50	57.100	16,631	162,824	0	33.33%
74.62%	5.96%	0.2500	5,424,164,957.30	31.990	10,916	139,780	0	42.86%
57.39%	8.77%	0.3800	3,152,519,439.70	57.100	16,631	162,824	0	37.50%
69.15%	1.38%	0.0300	2,657,002,804.19	46.650	33,611	499,167	0	33.33%

Ownership Concentration	Current liabilities	Total indebtedness	Corporate debt structure
45.40%	4,512,724,004.47	4,776,279,424.42	94.48%
46.53%	54,492,629,975.17	61,439,829,276.11	88.69%
23.47%	22,909,119,249.19	30,652,838,930.82	74.74%
32.33%	12,797,903,872.22	13,109,098,675.95	97.63%
62.86%	10,497,327,071.88	10,983,904,233.45	95.57%
36.48%	13,532,936,051.39	22,642,631,787.25	59.77%
30.41%	6,785,308,428.67	7,036,690,195.97	96.43%
59.22%	3,829,139,775.81	4,423,114,535.18	86.57%
33.39%	26,102,882,224.95	31,122,285,706.33	83.87%
29.28%	4,176,707,325.97	4,861,010,353.02	85.92%
29.83%	62,758,605,235.89	73,343,584,061.91	85.57%
20.77%	1,793,345,682.45	1,995,939,614.36	89.85%
46.51%	6,824,820,714.98	7,636,425,025.31	89.37%
22.37%	12,713,524,185.37	20,701,805,704.21	61.41%
25.78%	7,012,173,073.36	8,156,278,185.06	85.97%
32.32%	57,596,072,592.03	76,105,349,259.47	75.68%
8.26%	4,847,623,594.20	5,650,089,394.20	85.80%
23.38%	7,932,760,536.46	8,579,469,406.67	92.46%
44.45%	13,064,065,871.45	17,545,296,822.97	74.46%
51.13%	688,979,080,000.00	819,217,639,000.00	84.10%
47.21%	137,504,984,000.00	169,633,389,000.00	81.06%
55.10%	305,923,537,000.00	341,600,705,000.00	89.56%
56.30%	1,145,727,677,000.00	1,532,616,609,000.00	74.76%
58.34%	352,788,441,394.01	619,756,561,201.17	56.92%
45.39%	526,015,547,122.00	824,020,184,999.00	63.84%
34.79%	1,623,677,623.82	1,863,910,709.69	87.11%
24.48%	6,072,431,809.85	6,072,431,809.85	100.00%
24.39%	23,231,902,893.68	24,073,395,954.33	96.50%
31.19%	5,934,862,704.76	7,999,063,960.84	74.19%
32.77%	13,249,164,002.50	13,361,677,446.96	99.16%
39.71%	14,837,923,709.87	17,387,469,572.89	85.34%
20.15%	3,214,897,358.55	3,264,897,358.55	98.47%
39.30%	2,741,900,946.49	2,751,162,777.17	99.66%
28.07%	3,027,071,834.62	3,177,016,842.04	95.28%
41.66%	4,552,348,090.80	4,709,055,693.79	96.67%
34.15%	1,663,099,689.39	1,942,155,319.44	85.63%
41.99%	5,063,257,927.27	5,112,311,292.12	99.04%
27.35%	18,686,102,299.57	19,520,774,585.09	95.72%
31.06%	7,182,899,096.78	7,195,197,672.46	99.83%
35.42%	2,007,361,754.63	2,640,397,754.55	76.02%
37.93%	2,672,375,995.27	2,819,819,488.18	94.77%
48.88%	6,310,783,068.22	6,648,757,923.35	94.92%
46.53%	55,753,989,177.74	63,653,400,264.24	87.59%
23.47%	28,889,478,544.45	37,961,344,304.31	76.10%

32.33%	13,667,734,164.18	13,952,207,973.52	97.96%
62.86%	10,576,611,112.49	10,988,220,628.90	96.25%
36.48%	16,621,358,080.06	25,648,333,970.39	64.80%
30.41%	8,505,490,853.81	8,795,734,991.88	96.70%
58.12%	5,155,692,794.44	5,730,682,043.15	89.97%
31.84%	23,508,240,268.63	32,085,950,856.48	73.27%
29.28%	5,157,348,281.65	6,503,773,522.11	79.30%
37.90%	68,235,519,415.19	79,443,008,024.37	85.89%
20.77%	1,797,520,241.04	2,865,650,567.02	62.73%
46.51%	6,832,175,299.83	10,763,323,565.75	63.48%
17.10%	11,438,413,636.49	20,121,686,980.67	56.85%
32.78%	8,355,725,541.86	10,039,695,912.47	83.23%
32.32%	64,170,886,609.09	88,465,181,695.11	72.54%
8.26%	5,157,495,771.53	5,755,282,200.66	89.61%
23.38%	7,863,272,929.24	7,991,122,030.86	98.40%
44.45%	16,041,661,906.67	20,130,629,319.80	79.69%
51.13%	769,624,571,000.00	929,153,709,000.00	82.83%
47.21%	163,369,501,000.00	218,886,490,000.00	74.64%
49.18%	331,791,251,000.00	366,037,656,000.00	90.64%
56.31%	1,196,014,747,000.00	1,615,078,738,000.00	74.05%
58.34%	381,709,150,648.86	662,634,103,318.85	57.60%
57.99%	582,708,009,670.00	946,365,159,652.00	61.57%
35.21%	1,748,708,892.08	1,903,661,233.79	91.86%
22.00%	7,810,286,002.66	7,810,286,002.66	100.00%
23.69%	27,016,896,760.05	27,857,978,731.80	96.98%
31.18%	7,470,674,487.03	8,943,945,860.50	83.53%
32.77%	13,501,415,561.07	13,701,266,330.76	98.54%
39.71%	16,190,394,131.99	17,048,994,131.84	94.96%
20.17%	3,550,405,508.61	3,550,405,508.61	100.00%
29.99%	2,188,872,508.55	2,392,045,815.12	91.51%
24.40%	3,827,535,810.87	3,846,336,868.51	99.51%
41.66%	3,981,545,607.03	4,056,373,923.27	98.16%
28.78%	1,323,000,646.57	1,596,061,011.43	82.89%
42.01%	4,595,906,693.98	4,722,208,911.37	97.33%
27.35%	18,656,525,321.45	19,398,422,127.23	96.18%
31.07%	7,322,839,057.24	7,841,346,144.72	93.39%
35.42%	2,245,562,258.30	2,877,892,998.71	78.03%
29.76%	3,189,702,816.62	3,645,583,010.66	87.49%
